# A call centre and extended checklist for pre-screening elective surgical patients – a pilot study

**DOI:** 10.1186/s12871-015-0057-1

**Published:** 2015-05-19

**Authors:** Guy Ludbrook, Richard Seglenieks, Shona Osborn, Cliff Grant

**Affiliations:** 1 Discipline of Acute Care Medicine, The University of Adelaide, Adelaide, North Terrace 5005 Australia; 2 Department of Anaesthesia, Royal Adelaide Hospital, Adelaide, North Terrace 5000 Australia

**Keywords:** Anaesthesia, Preoperative assessment, Computer-assisted, CATI, Cost-benefit

## Abstract

**Background:**

Novel approaches to preoperative assessment and management before elective surgery are warranted to ensure that a sustainable high quality service is provided. The benefits of a call centre incorporating an extended preoperative electronic checklist and phone follow-up as an alternative to a clinic attendance were examined.

**Methods:**

This was a pilot study of a new method of patient assessment in patients scheduled for elective non-cardiac surgery and who attended a conventional preoperative clinic. A call centre assessment, using a Computer-assisted Health Assessment by Telephone (CHAT), paper review by an anaesthetist, and a follow-up phone call if the anaesthetist wished more information, preceded the conventional preoperative clinic. Summaries from the call centre and clinic assessments were independently produced.

The times spent by call centre staff were recorded. The ‘procedural anaesthetist’ (who provided anaesthesia for each patient’s actual surgery/procedure) documented an opinion on whether the call centre assessment alone would have been sufficient to bypass the preoperative clinic if the patient were hypothetically undergoing laparoscopic cholecystectomy. This opinion was also sought from a panel of four senior anaesthetists, based on patient summaries from both the call centre and preoperative clinic, but expanded to three hypothetical operations of different complexity – cataract removal, laparoscopic cholecystectomy, and total hip replacement.

**Results:**

Call centre assessment followed by clinic attendance was studied in 193 patients. The mean time for CHAT was 19.8 (SD 7.5) minutes and, after review of CHAT summaries, anaesthetists telephoned 45.6 % of cases for follow-up information. The mean time spent by anaesthetists on summary review and phone calls was 3.8 (SD 3.9) minutes. Procedural anaesthetists considered 89 % of the patients under their care suitable to have bypassed the preoperative clinic if they were to have undergone cholecystectomy. The panel of senior anaesthetists judged 95-97 % of patients suitable to have bypassed preoperative clinic for cataract surgery, 81-85 % for cholecystectomy and 79-82 % for hip replacement.

**Conclusions:**

A call centre to pre-screen elective surgical patients might substantially reduce patient numbers attending preoperative anaesthetic assessment clinics. Further studies to assess the quality of such an approach are indicated.

**Trial registration:**

ANZCTRACTRN12614000199617.

## Background

Obtaining an effective patient history is essential to the process of patient evaluation and management. This is true for preoperative evaluation by anaesthetists, an area where there is increasing evidence of associations between imperfect or absent assessment and either poor outcomes [1, 2] or poor use of resources through late case cancellations [3].

However, the combination of increasingly prevalent patient comorbidities and resource limitations challenge anaesthetists’ and healthcare organisations’ capacity for good preoperative assessment. To address this, a number of strategies have been trialled, including nurse-led clinics, videoconferencing, and internet questionnaires [4, 5]. Formal analysis of areas such as quality and cost is important to allow an evaluation of the overall value of such approaches.

Computer Assisted Telephone Interviews (CATI) have proven a valuable tool in areas such as collection of patient information in public health [6, 7]. In a similar vein, the authors have previously found a Computer-assisted Health Assessment by Telephone (CHAT) provides patient data of sufficiently high quality that the preoperative assessment could safely occur on the day of surgery in 60 % of cases in a tertiary care hospital, according to specialist anaesthetists participating in the study [8]. The potential impact on preoperative resource requirements of such an approach is substantial.

This study was designed to explore the benefits of adding call centre-based CHAT summary review by an anaesthetist, with anaesthetist-to-patient phone follow-up if needed. This was a pilot study looking at the utility of remote access pre-screening compared to standard care. The hypothesis was that call centre-based review of the CHAT summary by an anaesthetist would produce data of sufficient quality to have allowed a significant proportion of patients to be first seen on the day of surgery.

## Methods

This pilot study was approved by the Queen Elizabeth Hospital Human Research Ethics Committee. Data were collected prospectively between October 2012 and November 2013 at two metropolitan tertiary public hospitals in South Australia. As a pilot study of a change in practice, no formal attempt was made to power the study, but previous experience suggested recruiting approximately 200 patients would provide sufficient data on which to base future work.

This was a prospective study, with patient assessment via a call centre preceding the conventional outpatient clinic. Only the outpatient clinic visit was used for patient management. Study subjects were at least 18 years of age, with reasonable English comprehension, and attending anaesthetic outpatient clinic for workup before elective surgery. Subjects were approached by the booking proceduralist at the time they were scheduled for an elective procedure. They were provided with written information about the study and gave verbal consent to participate.

Patients received a call centre phone assessment 1 to 2 weeks before their scheduled preoperative anaesthetic outpatient appointment. After confirmation of their willingness to proceed, patients were asked a series of questions guided by a smart questionnaire and a computer. The general procedure for phone interviews using a computer-based smart questionnaire has been reported previously [8]. In brief, the interviewer was trained to follow the question script provided by custom-written computer software and used receptionist-level skills. The final question set was structured as a branching decision tree consisting of approximately 500 separate questions. Conditional questioning was used, so that primary questions might lead to follow-up questions to further clarify information provided, and so the total number of questions asked depended upon the patient's medical history. Answers were provided from a checklist of approximately 1500 fixed options, although limited free text could be added.

A summary of the CHAT was produced using dedicated software, with the layout mimicking existing preoperative assessment forms at each institution in order to assist clinicians to navigate and utilise the responses. The printed summary was reviewed by an anaesthetist in the call centre, who was called to attend when a number of summaries were available. The options available to the anaesthetist were (i) to consider this summary likely to be adequate for a patient to be seen on the day of surgery and sign it off, or (ii) to phone the patient to enquire further about some of the printed responses and then append the summary. The time taken for the initial phone call, and the time taken for the anaesthetist to review the summary and follow pathway (i) or (ii) were recorded.

After this call centre assessment, patients proceeded to their usual preoperative outpatient appointment, without the outpatient anaesthetist having access to the call centre summary. Both the call centre and outpatient clinic patient summaries were therefore independently generated, and the call centre assessments not used for patient management. These summaries were later reviewed by (i) the procedural anaesthetist performing anaesthesia for the patient, and (ii) a panel of four senior anaesthetists, consisting of the Directors of Anaesthesia of three metropolitan tertiary public hospitals and an academic anaesthetist. The procedural anaesthetists were asked whether the call centre assessment was sufficiently comprehensive to have allowed patients to be seen for the first time on the day of surgery, if they were hypothetically undergoing a laparoscopic cholecystectomy rather than their scheduled procedure. The panel was asked the same question, but the hypothetical procedures were expanded to include three surgical procedures of differing complexity: cataract extraction, cholecystectomy and hip replacement. These procedures were chosen as examples of the UK’s NICE grades of surgery. Reviewers were to assume that informed consent and preoperative investigations had been satisfactorily obtained. If reviewers deemed the CHAT assessment insufficient, reasons for a requirement to attend preoperative clinic rather than rely on the CHAT assessment alone were requested of all reviewing anaesthetists.

Patient demographic variables were collected from the outpatient assessments and entered into a database along with anaesthetists’ opinions. From the call centre, time taken for the CHAT assessment was recorded. Time taken to conduct CHAT summary review and follow up phone calls was recorded as the time spent by the reviewing anaesthetist. All data were pooled and presented as prevalence (%), mean (SD) or median (range), as appropriate.

In order to estimate the potential financial impact on the existing service, the time taken by clinical staff in the call centre and the existing preoperative clinic, along with hourly staff costs, were used to estimate a per patient cost of preoperative assessment. This was conducted for: (a) the existing model where the vast majority of patients scheduled for surgery involving overnight stay are seen in a clinic, and (b) a model where all were reviewed in a call centre, half were triaged to be seen on the day of surgery [8], and half were still seen, but in a streamlined clinic. For the purposes of this cost estimate, the time for assessment and management in these streamlined clinics was assumed to be half that of the conventional clinics, because of the phone pre-assessment, pre-ordering of necessary investigations, and pre-provision of appropriate patient information. Costs were based on the existing model at the Royal Adelaide Hospital, the tertiary hospital containing the call centre. This has approximately 23 operating suites and sees 8,000-12,000 patients annually in the preoperative clinic. It has a casemix similar to the patients in this study, and detailed data on current clinic resource requirements were available. No attempt was made to estimate indirect cost savings, such as patient travel, time taken from employment and infrastructure.

## Results

In total, 150 patients from each hospital received a call centre assessment. Because of cancelled and re-scheduled surgery, only 193 patients subsequently proceeded to the preoperative clinic. Of these, patients’ anaesthetists provided a judgement on the call centre summary on the day of their procedure for 178 patients. The panel reviewed all 193 cases.

Demographic data for these patients are shown in Table 1. The majority were ASA 2 and 3, with a mean (SD) age of 58 (15) years, and predominantly scheduled for endoscopic procedures. Obesity was common, with a mean (SD) BMI of 29.1 (7.9). Co-morbidities were frequent, with an incidence of hypertension, heart disease and diabetes of 47.2 %, 17.4 %, and 15.4 %, respectively.

**Table 1:** Patient characteristics

Characteristic	Value – incidence, mean or median
Male - %	51.8 %
Age – mean (SD)	58 (15) years
ASA 1	14.9%
ASA 2	54.4%
ASA 3	29.7%
BMI – mean (SD)	29.1 (7.9)
No. of medications – median (range)	4 (0–18)
Hypertension - %	47.2%
Heart disease - %	17.4%
Diabetes - %	15.4%
Surgery actually performed (number)	
- Endoscopy	135
- ENT/Ophthalmology	7
- Urology	12
- Gynaecology	17
- Vascular/Cardiology	2
- General surgery	12
- Orthopaedics	8

The mean (SD) time for initial CHAT phone calls was 19.8 (7.5) minutes. Anaesthetists who reviewed the CHAT summary in the call centre chose to call 45.6 % of cases. The topics requiring clarification on the phone varied greatly, but predominantly related to expansion of an issue ‘flagged’ by the self-assessment. Examples include further questioning about symptoms of dyspnoea or chest pain. The mean (SD) time spent on both paper and phone review of patients was 3.8 (3.9) min. Procedural and panel anaesthetists provided opinions on the proportion of cases whose call centre assessments were adequate to have hypothetically allowed the patient to have been seen first on the day of surgery. These are shown in Table 2. Procedural anaesthetists considered 89 % of the patients under their care would have been suitable to have bypassed the preoperative clinic if they were to have undergone cholecystectomy. The panel of senior anaesthetists judged 95-97 % of patients suitable to have bypassed preoperative clinic for cataract surgery, 81-85 % for cholecystectomy and 79-82 % for hip replacement.

**Table 2:** The proportion of surgical cases considered suitable to bypass the existing outpatient clinic if assessed in a call centre. The reviewers were the anaesthetist managing the case (the procedural anaesthetist) and a panel of four senior anaesthetists

Reviewer	Cataract	Cholecystectomy	Total hip replacement
Procedural anaesthetist		159/178 (89%)	
Reviewer 1	185/193 (96 %)	162/193 (84 %)	158/193 (82 %)
Reviewer 2	187/193 (97 %)	156/193 (81 %)	154/193 (80 %)
Reviewer 3	187/193 (97 %)	164/193 (85 %)	158/193 (82 %)
Reviewer 4	183/193 (95 %)	160/193 (83 %)	152/193 (79 %)

Procedural and panel anaesthetists decided a small minority of patients required a standard preoperative clinic assessment before the day of surgery, rather than a CHAT summary and day-of-surgery review. Their reasons are provided in Table 3. Staff were not specifically asked to justify this requirement, but, when provided, comments provided some insights. Of note, high BMI (usually > 50), cardiorespiratory disease, and known neck masses were common sources of concern for anaesthetists.

**Table 3:** Reasons for needing to attend outpatients. For some patients more than one reason was provided, and reasons were often not provided by procedural anaesthetists

Reason	Procedural anaesthetist	Panel	Comment
BMI	1	11	Panel cut off: BMI > 50
OSA	1	1	
Cardiac or Vascular disease	7	13	
Breathlessness		4	
Respiratory disease	1	10	
Neck mass or disease	1	4	Large thyroid
			Stiff neck
			Mouth opening
			Airway
Warfarin therapy		2	
Investigations	1		Cardiac report
Diabetes management	2	2	
Jehovahs Witness		2	Informed consent
Other		2	Previous ICU care
			Multiple diseases
			Cirrhosis
			Kidney transplant

Current clinical staff costs of standard preoperative clinic visits were estimated at AUS$295 per patient, based on award salary rates, and staff and patient numbers in each clinic. Estimated costs of a future model, with pre-review in a call centre and triaging of half to a streamlined clinic assessment and half to an assessment on the day of surgery, were AUS$157 per patient.

## Discussion

Remote patient interactions via telephone and videoconferencing may serve a range of purposes, including provision of patient advice, patient assessment, and triage to different care pathways. In settings other than elective surgery they have shown the potential to deliver benefits such as lower costs and improved access for rural and metropolitan patients [9–12], although the overall benefit to healthcare may depend on the context and the intent of the service offered [13–15]. The service proposed in the current study relates specifically to collection of detailed contemporary information from patients without the need for a face-to-face consultation with a medical specialist. Because of this specificity, and because of the known associations between imperfect preoperative assessment and outcomes [1, 2], careful analysis prior to any change in practice is warranted.

Telephone communication was chosen because of the simplicity and general ease of access to telephones, although the use of an on-screen checkbox approach for both questions and answers also lends itself to online use. However, since the capacity for patients to navigate this complex online questionnaire may depend on a number of factors, including familiarity with computers, and universal access to the internet is not certain, a phone operator with interpersonal skills verbally guided the patients through the questionnaire.

The call centre summaries were judged to be of sufficiently high quality to have allowed the vast majority of patients to bypass the preoperative clinic and be assessed by the anaesthetist on the day of surgery, if informed consent were effectively dealt with and relevant laboratory investigations were performed. This figure is higher than that of 60 % reported in a similar casemix in an earlier study [8]. This may relate to an enhanced question/answer set and to the clarification of specific anaesthetic issues provided by specialist phone follow-up. Whilst there remains a need to address elements such as informed consent, investigations and patient instructions, options other than an anaesthetic clinic visit exist for many patients. Further, even if clinic visits were required, their duration would be likely to be brief compared to the appointment durations currently scheduled, because much of the history is already obtained, investigation results assembled, and the process of informed consent commenced.

There are numerous potential cost benefits to remote access and pre-screening. Initial analyses of the impact on multidisciplinary public hospital preoperative clinics suggest a halving of per patient service costs. Furthermore, there may be reductions in missed appointments, time lost from work, and transport and infrastructure costs. A number of assumptions, however, are made. The impact on practice will depend on the patient population, casemix, and existing preoperative assessment methods, as well as access to contemporary medical records.

The basic cost analysis performed on this small cohort of patients includes assumptions which remain to be tested. Nevertheless, it justifies exploring this area further, and suggests this approach may also provide cost-effective options for centres where preoperative assessment is currently restricted because of access to staff or funding.

Remote pre-screening could also improve the quality of data collection. The benefits of checklists in medicine are increasingly recognised and, considering the time pressures on clinicians and an increasingly complex casemix, mandated questions and answers may act as an extended preoperative checklist to reduce the risk of clinicians overlooking uncommon events [16].

A significant limitation of the call centre pre-screening approach and bypassing preoperative clinic is the lack of physical examination before the day of surgery, and the risk of finding a late impediment to anaesthesia and surgery. For example, undiagnosed asymptomatic systolic murmurs have been reported at a rate of 15% in patients aged over 65 years [17]. Although the significance of many of these may be low, mechanisms to manage this in the elderly might include access to previous medical records, ensuring ECGs are performed ahead of surgery, having elderly patients and those with cardiac disease attend a fast-tracked preoperative clinic, and the availability of anaesthetist-performed focused transthoracic echocardiography on the day of surgery [18]. Similarly, undiagnosed difficult airways presenting on the day of surgery might be expected. Cancellations due to airway problems are considered unlikely, as obvious major difficulties (e.g. airway masses, very poor mouth opening) can often be identified via questioning. This is shown in Table 3 where on four occasions the reason given for a patient needing to attend a preoperative clinic related to possible airway issues. Further, modern advanced airway equipment is readily available in modern secondary or tertiary hospitals.

The risks presented by an absence of physical examination primarily relate to cancellations on the day of surgery, when face-to-face assessment will occur even for those not attending a clinic. It must be acknowledged that a large proportion of patients presenting for elective surgery in other Australian healthcare settings, such as the private health sector, are currently first seen by an anaesthetist on the day of surgery, often with much less detailed pre-screening systems in place.

There are some study limitations. An English-speaking subset of all patients was used, and excluded those considered unlikely to comprehend questions via telephone. Nevertheless, patient demographics were considered typical of elective surgical patients in the public sector, and were similar to the profile of patients treated in the recovery room of the Royal Adelaide Hospital, the largest tertiary hospital in South Australia [19]. Even if only a subset of patients can be effectively assessed in this manner, it represents a comprehensive mechanism for timely triage and streaming a large percentage of patients. Future translation services are feasible.

Many patients who received a call centre assessment in this study had surgery cancelled or deferred and did not attend preoperative clinic. Because clinic assessments were not available, the quality of call centre data could not be assessed. As these cancellations related to changes to surgery scheduling in a busy public health sector, rather than patient characteristics, the impact on the results presented here is considered to be small.

This is a pilot study, and further careful study is required before routine use of call centre pre-screening, although the challenges of statistical proof of superiority or non-inferiority for health services delivery are substantial. For example, analysis of an earlier study [8] suggests that a prospective randomised study to demonstrate non-inferiority on quality would require approximately 18,000 patients. Currently, a trial implementation of a call centre, using two fulltime non-clinicians making phone calls and assimilating other information, and clinicians reviewing phone summaries, is underway.

## Conclusions

Remote computer-assisted telephone pre-screening of elective surgical patients may have a place in delivery of accessible high quality sustainable preoperative assessment, but further studies are indicated to assess the quality of information obtained and its utility as part of a model of preoperative care.
